# FRCA: A Fuzzy Relevance-Based Cluster Head Selection Algorithm for Wireless Mobile *Ad-Hoc* Sensor Networks

**DOI:** 10.3390/s110505383

**Published:** 2011-05-18

**Authors:** Chongdeuk Lee, Taegwon Jeong

**Affiliations:** Division of Electronic Engineering, Chonbuk National University, Jeonbuk, Korea; E-Mail: cdlee1008@jbnu.ac.kr

**Keywords:** resource management and sharing, mobile *ad hoc*, clustering, fuzzy relevance, mobility, flat structure

## Abstract

Clustering is an important mechanism that efficiently provides information for mobile nodes and improves the processing capacity of routing, bandwidth allocation, and resource management and sharing. Clustering algorithms can be based on such criteria as the battery power of nodes, mobility, network size, distance, speed and direction. Above all, in order to achieve good clustering performance, overhead should be minimized, allowing mobile nodes to join and leave without perturbing the membership of the cluster while preserving current cluster structure as much as possible. This paper proposes a Fuzzy Relevance-based Cluster head selection Algorithm (FRCA) to solve problems found in existing wireless mobile *ad hoc* sensor networks, such as the node distribution found in dynamic properties due to mobility and flat structures and disturbance of the cluster formation. The proposed mechanism uses fuzzy relevance to select the cluster head for clustering in wireless mobile *ad hoc* sensor networks. In the simulation implemented on the NS-2 simulator, the proposed FRCA is compared with algorithms such as the Cluster-based Routing Protocol (CBRP), the Weighted-based Adaptive Clustering Algorithm (WACA), and the Scenario-based Clustering Algorithm for Mobile *ad hoc* networks (SCAM). The simulation results showed that the proposed FRCA achieves better performance than that of the other existing mechanisms.

## 1. Introduction

Wireless Mobile *Ad hoc* Sensor Networks (WMASNs) [1–3] are infrastructureless, multi-hop, dynamic networks established by a collection of mobile nodes. WMASNs consist of mobile sensor nodes that form the networks without any fixed infrastructure or centralized administration. In these networks, each node communicates with the other nodes immediately or via intermediate nodes. This kind of network is highly appealing due to its lack of infrastructure, cost effectiveness and simple installation. The considerations in these networks are to improve the network stability, scalability, bandwidth utilization, and resource sharing and management efficiency. Various clustering mechanisms are being applied to achieve these objectives [4–6].

Currently, clustering mechanisms are used for wireless mobile *ad hoc* networks in various areas, such as home networks, building automation, and ubiquitous applications. Clustering mechanisms are usually applied for large scale networks and thus involve high cost and overhead. Clustering strongly influences communication overhead, latency, congestion, inter-cluster and intra-cluster formation, as well as update policy. One of the solutions of the emerging problem is to cluster the distributed nodes in the flat structure or distributed network structure. The purpose of clustering in WMASNs includes stabilizing the network and routing, extremely sustaining bandwidth utilization and network effectiveness, minimizing energy consumption, and maximizing resource sharing and management. Therefore, an important point when dealing with clustering is how to create the clusterhead that plays an important role in cluster formation. The advantages of clustering include [2,7]:

- Shared use of the application within the cluster
- Provision for optimization in the routing mechanism
- Efficient handling of mobility management
- Spatial reuse of resources
- Better resource sharing and management
- Simplified routing scheduling
- Virtual circuit support
- Improved bandwidth utilization
- Aggregation of topology information
- Minimization of the amount of storage for communication

Typically, mechanisms utilized to overcome the overhead issue in WMASNs consist of the cluster-based algorithm, flat-based algorithm, and location-based algorithm [8,9]. The cluster-based algorithm divides the network size by a constant size. This algorithm creates the clusters using the divided network. However, creating the cluster via this algorithm is difficult because of the network size and dynamic property of mobile nodes. The flat-based algorithm is the routing approach based on flooding. This algorithm is based on routing the network addresses, while no data-driven routing is performed. The location-based algorithm decides the cluster using location information and residual energy power. This algorithm affects the problem of determining the lifetime of nodes in advance. Thus, if the nodes are managed in a distributed manner or flat structure without the cluster, the clustering performance is heavily affected by overheads.

Clustering mechanisms that divide a large scale network into several clusters are proposed to solve this kind of problem [10,11]. One of the first and most influential cluster-based algorithms is LEACH (Low-Energy Adaptive Clustering Hierarchy) [12], which uses a distributed probabilistic mechanism. Differently, the lowest-ID algorithm [9] constructs 1-hop clusters using the neighbor table that has information of the node ID, role of clusters, and link status (uni-/bi-directional) for nodes. This algorithm, however, generates too many cluster heads when the mobile *ad hoc* network grows or mobility increases. This algorithm selects cluster heads according to the strength of signal of nodes, and thus, the difficulty in accurate measurement of signal strength is another weak point of the algorithm. SCA (Secured Clustering Algorithm) [13] is a clustering algorithm that uses the trust value. This algorithm partially mitigates the cluster problems of 1-hop and 2-hop for clustering. Another algorithm, CBLARHM (Cluster Based Location-Aware Routing Protocol for Large Scale Heterogeneous Mobile *Ad hoc* Networks) [14], is based on GPS (Global Position System). This mechanism is utilized for clustering large scale networks, but involves high cost due to the use of GPS. These algorithms have difficulty in clustering and managing when the network size is variable.

To solve this problem, this paper proposes a novel Fuzzy Relevance-based Cluster head selection Algorithm (FRCA) that efficiently clusters and manages sensors using the fuzzy information of node status in the network. The proposed FRCA uses the Fuzzy Relevance Degree (FRD) with fuzzy value *μ* [15] to perform and manage clustering. We regard the Fuzzy Relevance Degree (FRD) with fuzzy value *μ* as FRD. Therefore, in this algorithm, FRD performs clustering by choosing some nodes that act as coordinators of the clustering. The fuzzy state viewing structure, which is performs clustering, consists of 5 parameters: ID, *μ*, Level, M-hop, and Balance. The cluster head ClusterHead (CH) and cluster members ClusterMember (CM) are selected using fuzzy value *μ* in the fuzzy state viewing structure.

In the proposed algorithm, FRD is used to solve expandability and to control the generation of multi-hop cluster. FRD controls the number of clusters to improve efficiency. The clustering based on FRD helps in maintaining the structure of the cluster as stable as possible, and thus minimizing the topology changes and associated overheads during ClusterHead changes. We compared the proposed algorithm with existing methods, such as CBRP (Cluster-Based Routing Protocol) [8], WACA (Weighted-based Adaptive Clustering Algorithm) [3], and SCAM [1] (Scenario-based Clustering Algorithm for Mobile *ad hoc* networks), in terms of performance. According to the simulation result, the proposed algorithm achieves better performance than the existing ones.

The rest of the paper is organized as follows. Related works are reviewed in Section 2. In Section 3, details of the proposed FRCA algorithm are presented. In Section 4, the simulation results of the proposed FRCA algorithm are given and the algorithm’s performances are discussed. Finally, in Section 5, some conclusions are drawn.

## 2. Related Works

Recently, several clustering algorithms were proposed to increase stability, routing performance, scalability, bandwidth utilization, and resource allocation in WMASNs. Clustering in WMASNs plays an important role in enhancing their basic network performance parameters like routing delay, congestion, energy consumption, and throughput. The hierarchical routing protocol in the clustering algorithm has been widely used for WMASNs. The existing flooding method [8–10] is the most popular hierarchical routing protocols. In this method, the source node communicates with the destination node irrespective of the movement speed.

In WMASNs, the number of control packets for flooding increases exponentially with the number of nodes. A number of clustering algorithms for WMASNs are proposed in the literature [16]. The CBRP (Cluster-Based Routing Protocol) methods were proposed to solve the problem of exponential increase [8]. The CBRP (Cluster-Based Routing Protocol) methods have been widely used to achieve efficient management and extension of distributed nodes. Well-known CBRP methods include LCA (Linked Clustered Algorithm) [17], LID (Lowest-ID) [9], LCC (Least Cluster Change) [18], MCC (Maximum Connectivity) [19], and RCC (Random Competition Clustering) [20]. These existing algorithms have clustering criteria for selecting cluster heads and are based on the minimum cluster overlap method in the formation of clusters [21,22]. These algorithms, however, cannot guarantee stability due to the ambiguity in the selection of cluster heads.

Thus, several clustering algorithms were proposed in WMASNs to improve performance and reduce overhead [23,24]. Selecting the cluster head is based on the mobility of nodes in [25], and on the mobility of nodes and power capacity in [26]. In [1], a scenario-based clustering algorithm (SCAM) was proposed, where (k,r)-dominating set was used for selecting the cluster heads and gateway nodes; here, k is the minimum number of cluster heads per node in the network, and r is the maximum number of hops between the node and the cluster head. This is to compute the quality of all dominating nodes. In [3] and [10], the clustering algorithms based on weighting were proposed, which considered link connectivity, power capacity and distance of nodes, and mobility in the selection of cluster head. These algorithms have the advantage of clear selection of the cluster head, but they have the problem of requiring correct information for the attributes and relationships of nodes. Though many clustering algorithms are proposed, few algorithms are dedicated for wireless mobile *ad hoc* networks.

The Lowest-ID method [9], one of the most popular methods for mobile *ad hoc* networks, has ambiguity in clustering due to selecting the cluster head with the lowest value. In [21], AMCS (Adaptive Multi-hop Clustering Scheme) was proposed as a wireless mobile routing algorithm. The AMCS algorithm reaches the destination node in multi-hop through the cluster head. This algorithm, however, has a problem in that the role of a node is not clear, whether it is the cluster head or the gateway, during the reception of local information from neighbor mobile nodes.

A centralized clusterhead selection algorithm was proposed in [27], where the base station assigned the cluster head roles based on the energy level and geographical position of the nodes. In [28], a centralized algorithm based on fuzzy was proposed, where the nodes were selected as cluster heads by the base station based on their distances from each other, energy level, and the concentration of nodes in the region. In [3], a distributed deterministic cluster head selection algorithm based on WCA (Weighted Clustering Algorithm) was proposed. WCA maintains 1-hop clusters with one clusterhead. The weight of each node is used in the selection of the cluster head. WCA considered geographical information and relative distances of nodes for the weight information. In [29], a distributed cluster head selection algorithm was proposed, where each node computes its priority based on its ID, current communication round, energy level, and speed. In this algorithm, the nodes with the highest priority become cluster head. In [16], a Topology Adaptive Clustering Algorithm (TACA) was proposed, where two major node parameters, like its mobility and battery power, were considered for achieving node suitability and cluster head. This improved the network life time and reduced maintenance overhead. In [3], a weighted-based adaptive clustering algorithm optimized for mobile hybrid networks (WACA) was proposed, where investigations focused on the problem of minimizing cluster head re-elections by considering stability criteria. These criteria were based on topological characteristics as well as on device parameters. This was to avoid needless cluster head re-elections for stable clusters in mobile *ad hoc* networks. However, the existing algorithms did not consider reliability, scalability, automatic awareness among cluster heads, clusterhead candidate and cluster member, dynamic change due to mobility, and the fuzziness of cluster head formation when the network size increased in proportion to the node’s number in flat structure or distributed network structure.

Thus, this paper proposes a Fuzzy Relevance-based Cluster head selection Algorithm (FRCA) to solve problems, such as energy consumption, transmission rate reduction, decrease in throughput, and incorrect cluster head election. The proposed FRCA constructs clusters more efficiently by reducing the incorrectness and ambiguity in the selection of cluster heads.

## 3. The Proposed Fuzzy Relevance-Based Cluster Head Selection Algorithm

This section describes the cluster head selection algorithm based on fuzzy relevance. The efficient formation of clusters plays an important role in the processing rate, performance improvement, and network stability.

### 3.1. Basic Clustering Concept

**Figure 1.**
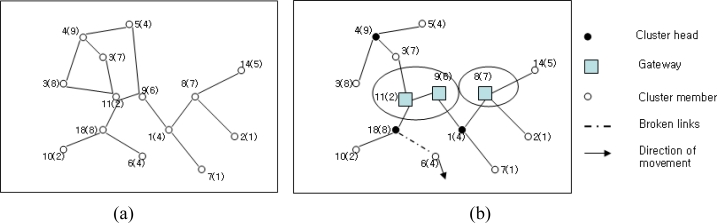
Flat structure and Hierarchical structure. (**a**) Flat structure. (**b**) Hierarchical structure.

Clustering in WMASNs can be considered as the virtual partitioning of dynamic nodes in the flat structure or distributed network structure into several clusters [30]. Clusters of the nodes in the flat structure or distributed network structure are made with respect to their nearness to each other. Such nodes are considered neighbors when all neighboring nodes are located within their transmission range and set up a bidirectional link between them. Typical algorithms for clustering in the flat structure or distributed network structure are known as one-hop clustering and multi-hop (d-hop) clustering algorithms [30]. In the one-hop clustering, every member node is at most 1-hop distance away from a central node that is called the cluster head. Thus, all member nodes remain at most two hops distance away from each other within a cluster category. On the contrary, in multi-hop clustering [21,30], the management of neighboring nodes to the cluster head is performed by allowing the nodes to be presented at most d-hop distance away from each other to form a cluster. A typical WMASN structure consists of flat and hierarchical structures as shown in Figure 1(a,b).

The small circle in the figure represents the nodes in WMASNs. The lines joining the circles denote connectivity among the nodes. Every node is identified with an ID number (*i.e.*, 1–14) along with a number within parenthesis. The numbers in the parenthesis are the weights of the nodes. These weights are measured with respect to various node parameters and apply the selection of clusterheads. Every node in the flat structure shares equal responsibility to act as a router to route the packets to every other node. However, to achieve better routing efficiency, this structure requires an amount of message flooding. Occasionally, such message flooding has the merit of reducing overhead of the MAC layer. On the other hand, nodes in the hierarchical structure are assigned with different functionalities while acting as a clusterhead, gateway, or a cluster member as shown in Figure 1(b). The clusterhead in the hierarchical structure plays an important role in inter-cluster and intra-cluster communication. Thus, the clusterhead works as the local coordinator for its member nodes and manages the cluster members. A gateway node is a node that connects the bridge between the inter-cluster and intra-cluster communication. A gateway works as the common or distributed access point for two cluster heads. Both of the distributed gateways provide the path for inter-cluster communication. The ordinary nodes of the cluster are the immediate neighbors of the cluster heads. They have the capability of serving as either a head or a gateway whenever selected to do so.

### 3.2. FSV Structure for Clustering

**Figure 2.**
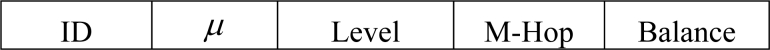
Packet structure of FSV.

FSV (Fuzzy State Viewing) structure clusters adaptively and is efficient when the size of networks varies according to the mobility of nodes. In the FSV structure, a node transmits not only packets but the fuzzy value [11] to neighbor nodes. The determined fuzzy value is used to prevent interferences and attacks from other nodes. A cluster is composed of a CH, CH candidate, gateway, and CMs, where CH is Cluster Head and CM is Cluster Member. Cluster nodes, classified as CH, CM, gateway node, and CH candidate according to their roles, broadcast packets shown in Figure 2 to neighbor nodes.

The parameters of the packet are explained as follows:

- *Identifier (ID)*: ID is assigned for identifying each node and used to avoid interference and attacks from other nodes during the selection of cluster head.
- *Fuzzy Relevance Degree ( μ )*: Fuzzy Relevance Degree (FRD) is a fuzzy value *μ* (0 ≤ *μ* ≤ 1), determined by available power, distance, and mobility. To reduce the computational complexity, we set *μ* a fuzzy value between 0 and 1, *i.e.*, ranging in {0, 0.1, 0.2, 0.3, 0.4, 0.5, 0.6, 0.7, 0.8, 0.9, 1}. FRD is used for selecting the cluster head and construction of clusters.
- *Level*: Each node has a level assigned according to the FRD of each node. Three levels are proposed: low level (Level-0) with *μ* ≤ 0.4, middle level (Level-1) with 0.5 ≤ *μ* ≤ 0.7, and high level (Level-2) with *μ* ≥ 0.8. The assigned levels are used in the selection of CH, CM node, and CH candidate nodes, and they are also are used to avoid the complexity of cluster management.
- *M-hop (Multi-hop)*: M-hops controls the management and generation of the 1-hop cluster and 2-hop cluster according to FRD. In large scale networks, the 1-hop cluster and 2-hop cluster generate too many cluster heads. Thus, the M-hops Adjustment adjusts the size of clusters according to the network size.
- *Balance*: Each cluster head is selected according to FRD (*μ*). The balance parameter is used to balance the number of nodes in clusters for achieving fair management of the attached cluster members (CM).

### 3.3. Cluster Head Selection

Efficient selection of the cluster head (CH) has a big influence on the cluster structure. This paper proposes the use of FRD to select the CH that is different from existing mechanisms such as CBRP [9], WACA [3], SCAM [1], and SCA (Secured Clustering Algorithm) [13]. The selection of the cluster head is complex and inaccurate in CBRP [9] based on Lower ID, MOBIC [25] based on mobility, and SCA (Secured Clustering Algorithm) [13] based on trust value. Existing mechanisms select the clusterhead using only one of the following parameters: ID, mobility, and trust value. The proposed method, however, uses parameters jointly to select the cluster head, and the cluster head is selected by FRD and determined by the available power, signal strength, and distance between the nodes, which is presented as follows.

#### 3.3.1. Fuzzy Relevance Degree

Fuzzy Relevance Degree (FRD) of a node represents the degree of reliability provided by neighbor nodes in the network. The FRCA system proposed in this paper selects the cluster head based on the fuzzy relevance, available power of nodes, mobility, and the distance between nodes. The available power of nodes, distance between nodes, and mobility of nodes are considered to maintain the balance of energy consumption of the nodes. The distance between nodes and mobility is considered to keep the balance between clusters. The FRCA performs clustering based on parameters described above and selects the cluster head for efficient clustering.

For n nodes of N={x_1_, x_2_, ..., x_n_}, the fuzzy set, *μ* (*x_i_*), is defined by the following Equation (1):

(1) $$\mu (x_{i})=\{\mu (x_{1}),\;\mu (x_{2}),\ldots ,\mu (x_{n})\},\;\;(1\leq i\leq 1)$$

Here, *x_i_* is a member node for clustering in the network, and *μ* (*x*) is a membership function. Then, the fuzzy relevance degree for node *x_i_*, *FRD*(*x_i_*), is defined by the following Equation (2):

(2) $$FRD(x_{i})=\frac{E_{i}(t)}{\sum_{j=1}^{n}E_{j}(t)}\times \mu (x_{i}).$$

where *E_i_* (*t*) is the energy of node *x_i_* at time t given by the sum of the available power of neighbor nodes for node *x_i_*. For example, assume that nodes *x*_2_, *x*_4_, *and x*_5_ are neighbor nodes of *x*_10_, and *p*_2_, *p*_4_, *and p*_5_ are the available powers of neighbor nodes *x*_2_, *x*_4_, *and x*_5_, respectively. Then, we get *E*_10_(*t*) = *p*_2_ + *p*_4_ + *p*_5_.

#### 3.3.2. Available Power

The available power is the available energy capacity. In this paper, we consider the energy power level of each node while calculating the available power, in order to increase the network lifetime. Whenever a node forwards a packet, it loses some amount of energy whose amount depends on factors such as the nature of packets, their size, access frequency, and the distance between the nodes. An available power function considers all these factors and decides which one, among all the discovered paths, should be selected for an energy-efficient transmission. We have considered individual energy power in considering the path, that is, if there is a path with a node having very low energy level, then the available power function does not select that path, irrespective of whether or not that path is time efficient.

The available power for node *x_i_*, *AP*(*x_i_*), depends on the number of nodes for the cluster i. The larger *AP*(*x_i_*) means the more stable power and the more energy power. Thus, the node with large *AP*(*x_i_*) is highly likely to be selected as a cluster head and able to support the network lifetime for a long time. Therefore, we consider the available power function to increase the network lifetime, and it is defined by Equation (3):

(3) $$AP(x_{i})=\frac{\sum_{x_{j}\in {\text{Cluster}}_{i}}P_{x_{j}}}{n_{i}}$$

where *n_i_* is the number of nodes in the cluster *i*, and *P_x_j__* is the available energy power of the node *x_j_*.

#### 3.3.3. Signal Strength

We denote *RS*(*x_i_*) as the received signal strength of node *x_i_* Typically, the signal strength between the sender and receiver depends on the physical distance between the nodes, and it is shown as *d_x_i_, x_j__* [11], where *d_x_i_, x_j__* is the distance between the cluster i and the member node j. However, in the real *ad hoc* network, the measured signal strength is ambiguous and inaccurate due to the dynamic mobility. This ambiguity and inaccuracy have a negative effect on the selection of cluster head.

Here, the signal strength based on FRD is introduced to solve the problems issued from the ambiguity and inaccuracy in the signal strength of member node j with respect to the cluster i. $FRD_{i}^{\mu }(x_{i})$ represents the relevance degree of the signal strength from the cluster i to member nodes and obtains the relevance information according to the signal strength between the cluster i and member node. The received signal strength function for node *x_i_*, *RS*(*x_i_*), is to measure the signal strength ratio of the cluster head and member nodes, which is defined by Equation (4):

(4) $$RS(x_{i})=10{\text{log}}_{10}\frac{\sum_{x_{j}\in \{\text{neighbor}\;\text{nodes}\;\text{of}\;x_{i}\}}FRD(x_{j})}{FRD(\text{clusterhead}\;\text{of}\;x_{i})}$$

#### 3.3.4. Distance

The distance between the cluster head i and member node j, *d_x_i_, x_j__*, is determined by the number of hops for the shortest path. Thus, the cost for distances of nodes in the cluster is an important factor. The distance cost between nodes is measured from the cluster head to member nodes. Here, the distance for the clusterhead *x_i_* is defined by the Equation (5):

(5) $$d(x_{i})=\sum_{x_{j}\in \{\text{cluster}\;\text{members}\;\text{of}\;x_{i}\}}d_{x_{i},\;x_{j}}$$

#### 3.3.5. Join

We measure CH based on the available power, signal strength, and distance mentioned above. Considering the available power, signal strength, and distance, the joint metric is defined by Equation (6):

(6) $$\text{Cost}(x_{i})=AP(x_{i})+RS(x_{i})-d(x_{i})$$

We calculate *Cost*(*x_i_*) for all potential cluster heads, and we then select the cluster head with the minimum *Cost*(*x_i_*).

**Figure 3.**
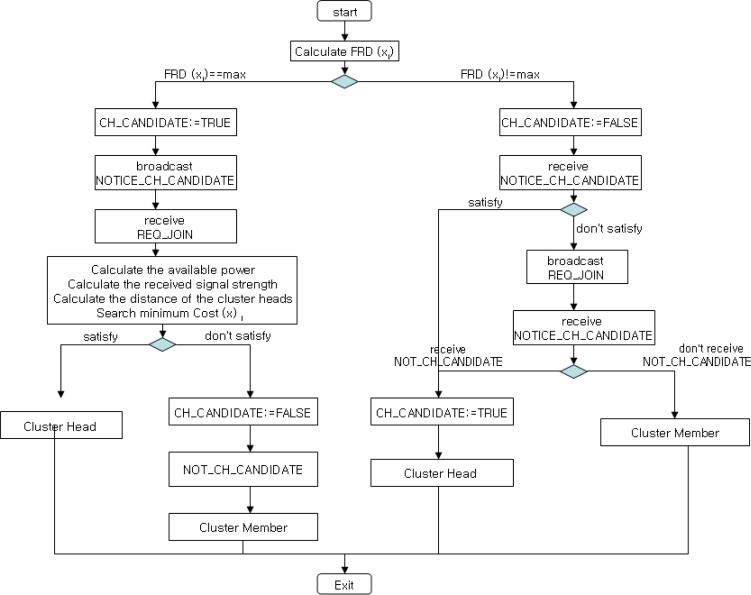
Flowchart for cluster head selection.

First, a node with more energy power and stronger signal has more probability to be the cluster head in a cluster. Thus, the node with the minimum cost becomes the cluter head candidate. Second, a non-cluster head node with higher energy power than those of neighbor nodes may become a cluster head candidate. The selected cluster head candidate has to notify its neighbor nodes of cluster head candidate selection (NOTICE_CH_CANDIDATE). Third, cluster members that are not the cluster head broadcast join request message (REQ_JOIN) to the nearest cluster head. If a node is not the cluster head candidate (NOT_CH_CANDIDATE), then the node forwards to neighbor nodes that the node is a cluster member. The whole process is shown in Figure 3, and the corresponding cluster head selection algorithm (Algorithm 1) is given as follows.

**Algorithm 1.** Cluster head Selection.

Input: Nodes’ information in a Node Cluster
Output: CH Node
begin
broadcast E_i_ in Cluster Radius
receive E_j_ in Cluster Radius
$$FRD(x_{i})=\frac{E_{i}(t)}{\sum_{j=1}^{n}E_{j}(t)}\times \mu (x_{i})$$
If (FRD(*x_i_*)==max(FRD(*x_j_*) |j=1,2,…,n)) then begin
broadcast NOTICE_CH_CANDIDATE(i) in Cluster Radius
receive REQ_JOIN(i,j) in Cluster Radius
Cluster(i)=Cluster(i) ∪ {j}
calculate the available power
calculate the received signal strength
calculate the distance for the cluster heads
search min *Cost*(*x_i_*) = *AP*(*x_i_*) + *RS*(*x_i_*) −*d*(*x_i_*)
if (i!=j) then begin
send NOT_CH_CANDIDATE
end
else
CH_CANDIDATE=FALSE;
receive NOTICE_CH_CANDIDATE(j) in Cluster Radius
CH(i)=CH(i) ∪ {j}
if (CH(i)!=Ø) then begin
broadcast REQ_JOIN(i,j)
else
CH_CANDIDATE=TRUE;
end
end
end

### 3.4. Cluster Formation

**Figure 4.**
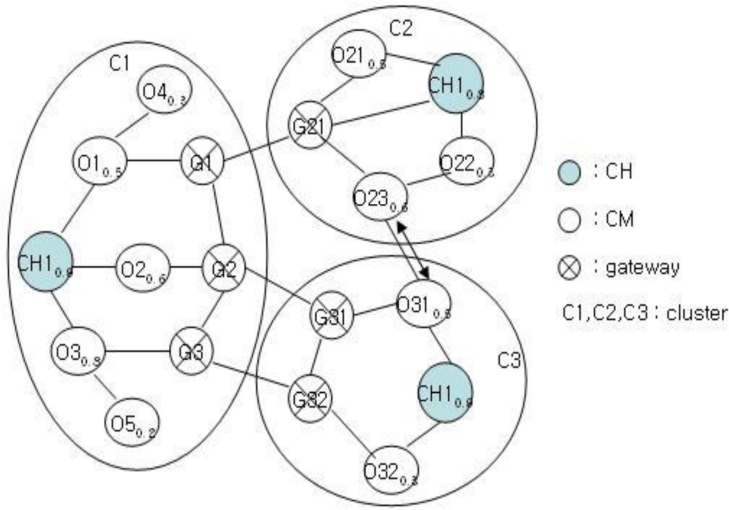
Original Cluster Structure.

After selecting CH by FRD, each cluster structure performs clustering for neighbor nodes. If a node needs clustering, then it checks the state of self-node first and checks the number of nodes of each cluster. Clustering is determined after checking the number of nodes by broadcasting the FSV packets. Let’s assume the cluster structure shown in Figure 4.

**Figure 5.**
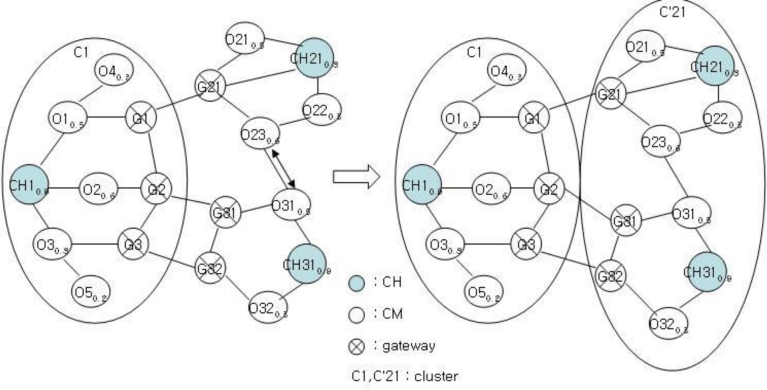
Modified Cluster Structure.

Figure 5 shows the cluster structure after the clustering of the structure in Figure 4. Each cluster of C1, C2, and C3 has a structure with a CH, gateway, and CM nodes. Clustering is performed for C2 and C3 to balance with C1. This clustering is very important in the proposed mechanism. The clustering of C1 and C2 or that of C1 and C3 results imbalance. After clustering, the clustering information is stored as shown in Tables 1 and 2 for achieving stable management and performance improvement of clusters.

After clustering, the existing cluster structure of Figure 4 is modified as shown in Figure 5, and the CH is to be changed. As shown in Figure 5, the nodes CH31 and CH21 become the new CH and the CH candidate, respectively.

## 4. Simulation Results

The paper used the NS-2 simulator [31] for the simulation to show the performance of the proposed method. In the simulation, the parameter values are selected at random and shown in Table 3. The parameters are network size, number of nodes, max speed, pause time, *μ*, packet size, transmission area, hello packet interval, and simulation time. The proposed method is compared with CBRP [9], WACA [3] and SCAM [1] for performance evaluation.

In the clustering mechanism, the generation of optimal number of clusters is very important to reduce the overhead and improve performance. Thus, the following five scenarios are considered to know the performance of the modified clusters.

- *Simulation Scenario 1*: The simulation is performed to evaluate the performance with the varying number of cluster heads. In the simulation, the number of nodes is 80, 160, 240, 320, and 380.
- *Simulation Scenario 2*: This scenario is to estimate the overhead according to fuzzy relevance degree *μ*. The simulation is performed for *μ* of 0.5, 0.6, 0.7, 0.8, and 0.9.
- *Simulation Scenario 3*: This scenario is for generating the cluster head according to the fuzzy relevance degree *μ*. The simulation is performed for *μ* of 0.5, 0.6, 0.7, 0.8, and 0.9.
- *Simulation Scenario 4*: This scenario is for testing CHER (ClusterHead Election Ratio). CHER depends on the network size. The simulation is performed for network sizes of 350, 400, 450, 500, 550, 600, 650, and 700.
- *Simulation Scenario 5*: This scenario is for the number of clusters with transmission range 200 m. The transmission range varies between 10 and 90 with a fixed step of 10. We were set to *μ* = 0.8 and *μ* = 0.9.

**Figure 6.**
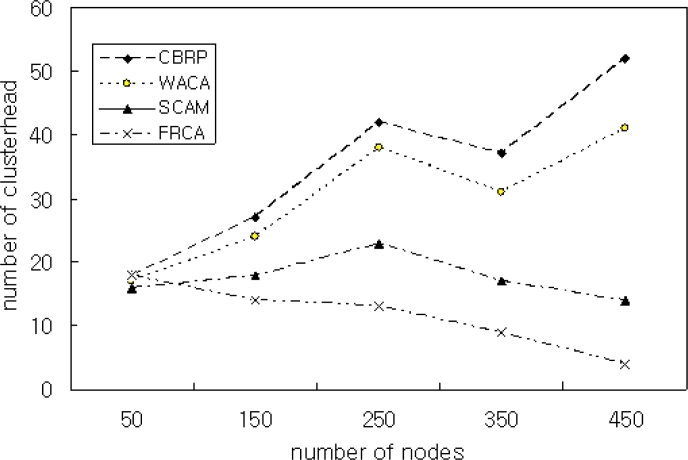
Number of clusterheads with the number of nodes N = 450 and *μ* = 0.9.

Figure 6 shows the simulation result for comparing CBRP, WACA, SCAM, and the proposed FRCA when the number of nodes is increased from 80 to 380. The simulation result shows that the proposed method has almost the same number of cluster heads as that of the other methods when the number of nodes is 80. As the number of nodes is increased, however, the proposed FRCA generates less cluster heads than the other methods. This means the proposed FRCA maintains the network performance efficiently by restricting the number of cluster heads.

**Figure 7.**
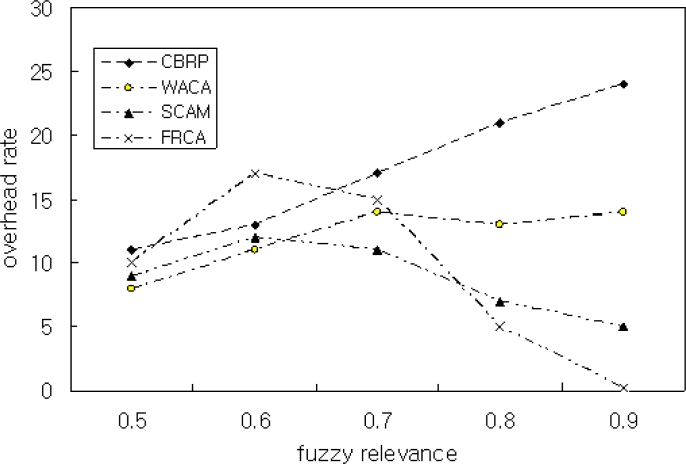
Overhead Rate with 0.5 ≤ *μ* ≤ 0.9.

Figure 7 shows the simulation result by Scenario 2 for the relation between overhead rating and FRD. The overhead rating of the proposed FRCA is similar to those of other methods when FRD(*μ*) is 0.5. This resulted from the fact that nodes are rated as CM when *μ* ≤ 0.7. The overhead rating is very low when *μ* = 0.9. In the simulation of the proposed method, there are only two overhead packets during the transfer of 220 packets when *μ* = 0.9. Thus, the use of FRD improved the throughput and performance and maintains clusters’ stability.

**Figure 8.**
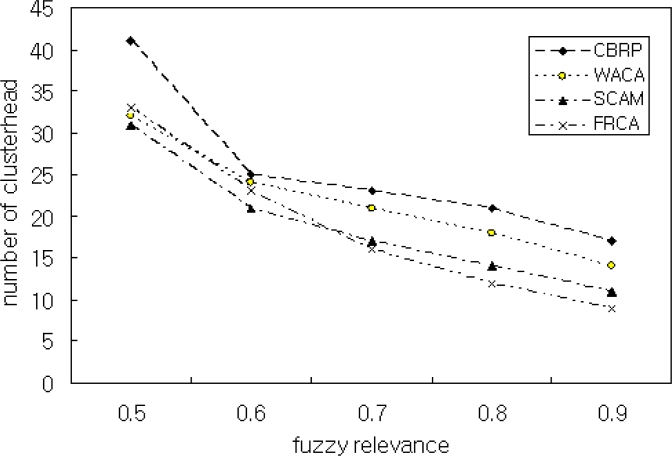
Number of Clusterheads with 0.5 ≤ *μ* ≤ 0.9.

Figure 8 is the simulation result for Scenario 3 and shows the relation between the number of cluster heads and FRD(*μ*). As shown in the figure, our method generated more cluster heads than the other methods when *μ* = 0.5. The reason for this is that our method generates cluster heads assuming *μ* ≥ 0.8. Therefore, the proposed FRCA generates the optimum number of cluster heads when *μ* ≥ 0.8. Too many cluster heads in clustering results difficulties in the management of clusters. In this paper, we assumed that a cluster head manages optimally about 100 nodes according to our experience. The simulation generated 4 clusters. The processing rate may be improved by adjusting the number of nodes in a cluster.

**Figure 9.**
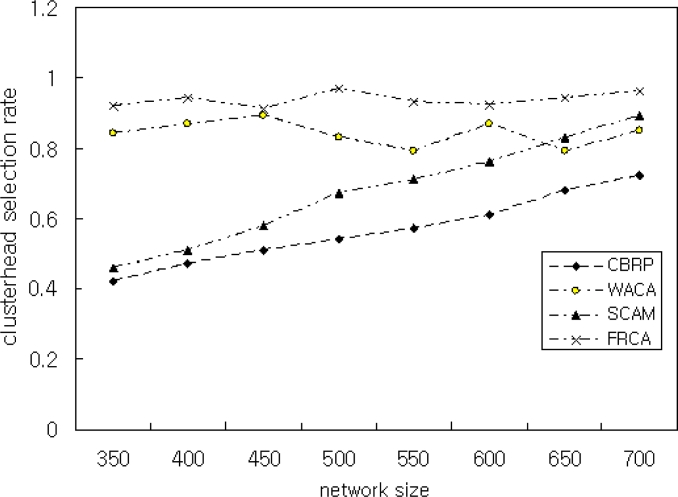
Clusterhead Selection Ratio with Network Size 700 and *μ* = 0.9.

In Scenario 4, we showed the performance of the proposed FRCA by varying network sizes. To achieve this, we vary the network size by 350, 400, 450, 500, 550, 600, 650, and 700. The simulation result is shown in Figure 9. As shown in Figure 9, the proposed FRCA achieves better CHER than SCAM that is known for its good performance. Better CHER of the proposed FRCA is due to the classification of nodes as the CH node, CH candidate node, or CH member nodes. Thus, the performance of the proposed FRCA does not degrade with the increase of network size. CHER is influenced by nodes with *μ* < 0.8 that means low signal intensity and low battery power. Therefore, the simulation is performed with FRD *μ* ≥ 0.9.

**Figure 10.**
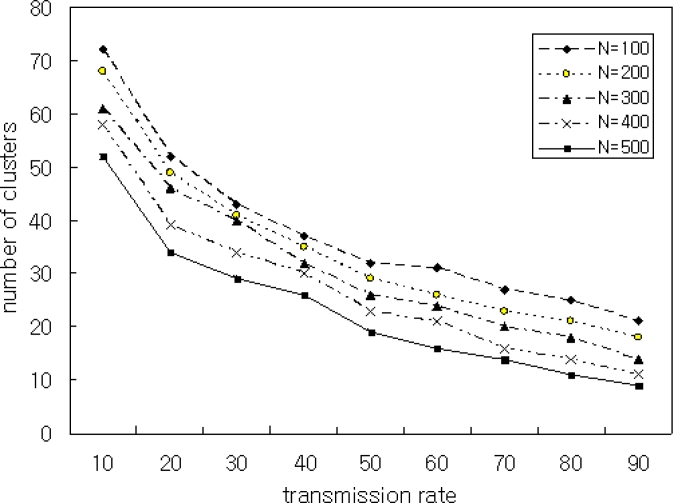
Transmission Rate *vs.* Number of Clusters with *μ* = 0.9.

**Figure 11.**
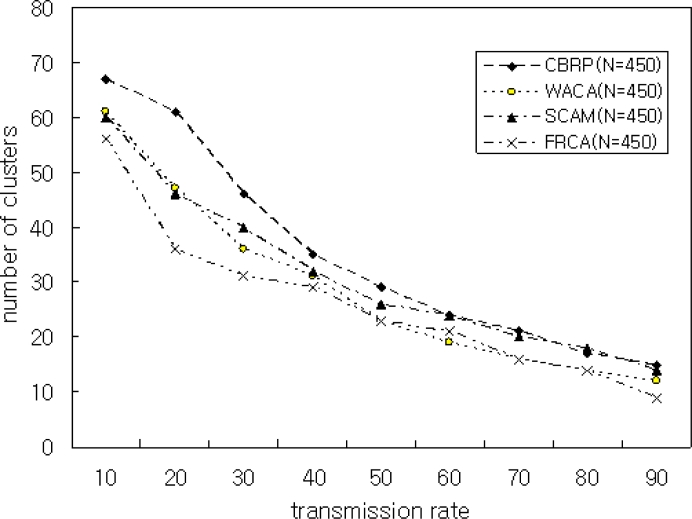
Number of Clusters in CBRP, WACA, SCAM, and FRCA with N = 450 and *μ* = 0.9.

In Scenario 5, we simulated the number of clusters by varying transmission ranges. To achieve this, we varied the transmission range between 10 and 90, and we varied the number of nodes N by 100, 200, 300, 400, and 500. The simulation results are shown in Figures 10 and 11.

Figure 10 shows the average number of clusters is relatively high when the transmission range is small. On the other hand, when the transmission range increases, the number of clusters created decreases. A smaller backbone reduces the routing overhead. Therefore, the transmission power of a node in a heterogeneous environment depends on the quality of dominating nodes. Figure 11 shows the simulation result with the number of nodes N = 400 and *μ* = 0.9. The proposed FRCA creates fewer clusters compared with those of CBRP, WACA, and SCAM. This is because the proposed FRCA applies FRD(*μ*) and results in form fewer clusters. But if FRD(*μ*) decreases more and more, then the cluster number and size decrease in proportion to FRD(*μ*), which affects the performance. Therefore, FRD(*μ*) is important to select the cluster head. Thus, the proposed FRCA selects the cluster head stably by filtering out nodes with low signal intensity and low battery power using the proper FRD(*μ*).

## 5. Conclusions

During the set up of routing in a wireless *ad hoc* network with mobile nodes, clustering is an important mechanism to build a stable network structure and to reduce the overhead and the table size. In case of large scale flat structure network environment, the overhead is due to the increase of management cost, the decrease in routing performance, the early consumption of battery energy, and the increase in the complexity of head selection.

This paper proposed a method, FRCA, to reduce the overhead. The proposed method used FRD for efficient selection of the CH and FSV for efficient clustering in the network. The proposed FSV is used to classify nodes under clustering as the CH node, the CH candidate, a gateway node, and CM nodes. For the efficient selection of the CH, existing methods used single measured parameter while the proposed method considered parameters such as FRD(*μ*), AP (*x_i_*), RS (*x_i_*), and *d* (*x_i_*).

The consideration of various parameters in the selection of CH node reduced the overhead due to the flat structure by easy resources management and bandwidth allocation, efficient management of node positions and energy, and the improvement of routing performance. The performance of the proposed method is compared with those of CBRP, WACA, and SCA with various combination of the number of nodes, fuzzy relevance degree, and the network size. The simulation result shows that the proposed method is more efficient than the other methods such as CBRP, WACA, and SCA.

## Figures and Tables

**Table 1.** Information for C1.

**Node**	**State**	**(***μ***)**

CH1	CH	0.9
G1	Gateway	
G2	Gateway	
G3	Gateway	
O1	CM	0.5
O2	CM	0.6
O3	candidate	0.8
O4	CM	0.3
O5	CM	0.2

**Table 2.** Information for C2.

**Node**	**State**	**(***μ***)**

CH21	candidate	0.8
CH31	CH	0.9
G21	Gateway	
G31	Gateway	
G32	Gateway	
O21	CM	0.5
O22	CM	0.3
O23	CM	0.6
O31	CM	0.5
O32	CM	0.3

**Table 3.** Simulation parameters.

**Parameters**	**Value**

Network Size	700 × 700
Number of Nodes	450
Speed	3–30 m/s
Pause Time	0 s
*μ*	0.5 ≤ *μ* ≤ 0.9
Packet Size	100 byte
Transmission Range	20–200 m
Simulation Time	420 s
Hello Packet Interval	3 s
MAC Protocol	IEEE 802.11
